# ASH2L induces tamoxifen resistance via H3K4me3 dependent ITGA6/ERK signaling in ER-positive breast cancer

**DOI:** 10.1038/s41416-026-03347-8

**Published:** 2026-02-24

**Authors:** Young-Hyeon Kye, So-Jeong Moon, Hea-Ry Cha, Tack-Hoon Kim, Jeong-Yun Eom, Jae-Kyung Myung, Gu Kong

**Affiliations:** 1 Department of HY-KIST Bio-convergence, College of Medicine, Hanyang University, Seoul, Republic of Korea; 2 Medicinal Materials Research Center, Korea Institute of Science and Technology, Seoul, Republic of Korea; 3 Department of Pathology, College of Medicine, Hanyang University Hospital, Seoul, Republic of Korea; 4 Department of Pathology, College of Medicine, Hanyang University, Seoul, Republic of Korea

**Keywords:** Breast cancer, Oncogenes, Cancer stem cells

## Abstract

**Background:**

Tamoxifen resistance remains a significant obstacle in oestrogen receptor (ER)-positive breast cancer. The function of absent, small, or homeotic 2-like protein (ASH2L) at chr8p11.23 in breast cancer is not entirely understood.

**Methods:**

Survival analysis according to ASH2L expression was examined using METABRIC (*n* = 968) and KM plotter (*n* = 150). ASH2L-mediated tamoxifen resistance and CSC activity were evaluated through in vitro assays, including SRB, colony formation, tumour sphere formation, and FACS, and in vivo xenograft models. RNA-seq and ChIP-qPCR were performed to elucidate the underlying mechanism.

**Results:**

High ASH2L amplification correlated with poor prognosis in tamoxifen-treated ER-positive breast cancer patients. ASH2L induces tamoxifen resistance and promotes CSC activity through ITGA6/ERK signalling in an H3K4me3-dependent manner. Mechanistically, ASH2L is recruited to HIF2A and ITGA6 promoters, enhancing H3K4me3 and H3K27ac and reducing HDAC1 and H3K27me3, thereby activating ERK signalling. Genetic or pharmacological inhibition of ASH2L, ITGA6, or ERK abolished ASH2L-induced CSC activity. Although ERK inhibition alone did not rescue tamoxifen resistance, its combination with tamoxifen overcame resistance in vitro and in vivo.

**Conclusions:**

ASH2L promotes tamoxifen resistance and CSC activity through ITGA6/ERK signalling. Combination therapy with tamoxifen and an ERK inhibitor may be a promising strategy for ER-positive breast cancer patients with ASH2L overexpression.

## Introduction

Breast cancer is the most common cancer and the main cause of cancer-related deaths in women. It is a heterogeneous disease with different biological subtypes. Oestrogen receptor (ER)-positive breast cancer is the most predominant breast cancer subtype, accounting for 75% of all breast cancers, and is a prototypical case of targeted therapy [1–3]. Tamoxifen is an important endocrine therapy for ER-positive breast cancer among the endocrine therapies [4]. However, tamoxifen resistance promotes cancer progression and results in poor prognosis in ER-positive breast cancer patients [5]. Resistance mechanisms include ER loss or mutations, as well as activation of the MAPK and PI3K/AKT signalling pathways [2, 6]. Therefore, it is crucial to identify novel targets to overcome tamoxifen resistance in ER-positive breast cancer.

The trithorax group (TrxG) protein, an absent, small, or homeotic 2-like protein (ASH2L), is required for complex-dependent methylation of histone H3 on lysine 4 (H3K4me) in mixed lineage leukaemia (MLL) [7]. ASH2L directly binds a GC-rich DNA motif using its helix-wing-helix (HWH) domain [8]. MLL complexes contain a methyltransferase (MTase) and core subunits consisting of the WD repeat domain (WDR5), retinoblastoma-binding protein 5 (RbBP5), ASH2L, and Dumpy-30 (DPY30), collectively referred to as the WRAD complex [9–11]. ASH2L is amplified or overexpressed in various human cancers, including breast cancer, and it promotes proliferation, epithelial-to-mesenchymal transition, metastasis, and angiogenesis [12–14]. However, the functional role of ASH2L in breast cancer tumorigenesis, including tumour initiation and progression, remains unclear.

Dysregulation of epigenetic regulation is closely associated with cancer pathogenesis, diagnosis, and treatment failure. This epigenetic regulation includes post-translational histone modifications, such as acetylation, deacetylation, and methylation, which regulate gene expression at the transcriptional level [15]. Recently, growing evidence suggests that histone methyltransferases (HMTs) and histone demethylases (HDMs) play crucial roles in the regulation of cancer stemness and progression. Among the histone marks, H3K4, H3K36, and H3K79 are active, whereas H3K9, H3K27, and H4K20 are suppressed at the transcriptional level [16]. Furthermore, accumulating evidence has revealed that these histone modifications cause anticancer drug resistance with poor prognosis, suggesting novel therapeutic targets for cancer treatment [17]. Nevertheless, the relevant biological mechanisms of histone modifications in terms of precision-targeted cancer therapy remain to be elucidated.

In the present study, we investigated the oncogenic role of ASH2L and identified a novel biological function related to tamoxifen resistance and cancer stem cell (CSC) activation in ER-positive breast cancer. We found that ASH2L regulates the hypoxia-inducible factor alpha (HIF2A)-integrin subunit alpha 6 (ITGA6)-MAPK signalling pathway in an H3K4me3-dependent manner to induce CSC-like properties and tamoxifen resistance in ER-positive breast cancer. These findings suggested that ASH2L is a potential therapeutic target for tamoxifen resistance in ER-positive breast cancers.

## Methods

### Survival analysis and public data

The survival of breast cancer patients (OS and DFS) according to ASH2L expression were analysed in the METABRIC and Kaplan-Meier Plotter (https://kmplot.com/analysis/). The patients were stratified by the ASH2L mRNA levels based on the optimised cut-off point maximising the *p*-value of significant difference between the groups. The hazard ratio of death according to the expression of genes on chromosome 8q11.23 in the METABRIC was analysed using Cancer Target Gene Screening (CTGS; http://ctgs.biohackers.net). Genetic alterations, copy numbers, and expression patterns of ASH2L in the METABRIC and TCGA datasets were analysed using cBioportal (http://www.cbioportal.org/) or CTGS. Datasets for H3K4me3 ChIP-seq (GSM945269 and GSE77772), TamR array (GSE14986) were downloaded from the GEO and were reanalysed using the Python language for comparison with our RNA-seq data.

### Colony formation assay

MCF7 (2 × 10^3^ cells/well), T47D (3 × 10^3^ cells/well), MCF7-TamR (1 × 10^3^ cells/well), and ZR-75–1 (4 × 10^3^ cells/well) were seeded in 24-well plates and cultured in DMEM or RPMI containing 10% FBS (Welgene). Cells were treated with 4-hydroxytamoxifen and/or SCH772984 for 6 days, followed by replacement with fresh DMEM or RPMI containing 10% FBS for an additional week. After PBS washing, cells were fixed with methanol (5 min, 4 °C), washed with sterile distilled water, and stained with 0.04% crystal violet (C0775; Sigma-Aldrich) for 30 min at room temperature. After destaining with sterile distilled water, staining intensity was quantified using the Colony Area plugin in ImageJ software (National Institutes of Health, Bethesda MD—USA) as previously described [18].

### Flow cytometry analysis

ALDEFLUOR experiments were performed following the manufacturer’s instructions to analyse cell populations with high ALDH enzymatic activity, a feature of CSC-like cells (Stem Cell Technologies, Cambridge, MA, USA). Briefly, 1 × 10^6^ cells were treated with 1 ml of ALDEFLUOR test buffer containing the ALDH substrate BODIPY aminoacetaldehyde (BAAA) at 37 °C for 20–60 min. For each sample in the experimental groups, diethylaminobenzaldehyde (DEAB), an inhibitor of ALDH, treated cells served as negative controls. Cell viability and the negative controls served as the basis for the sorting gates, and a FACS Canto Ⅱ was used to assess the ALDEFLUOR-positive population (BD Biosciences). For the analysis of breast CSC-like cell populations (CD44+/CD24-/ESA+ cells), cells were co-stained with allophycocyanin (APC)-conjugated CD44 (559942; BD Biosciences, San Jose, CA, USA), phycoerythrin (PE)-conjugated CD24 (555428; BD Biosciences), and fluorescein isothiocyanate (FITC)-conjugated ESA antibodies (60147FI; StemCell Technologies, Vancouver, BC, Canada), and analysed using a FACS Canto II flow cytometer (BD Biosciences) as previously described [19]. Apoptosis was assessed using the FITC Annexin V Apoptosis Detection Kit with Propidium Iodide (PI) (BioLegend) according to the manufacturer’s instructions. Briefly, 1 × 10^5^ cells were harvested, washed with cold PBS, and resuspended in 100 µL of binding buffer. Cells were then incubated with 3 µL of FITC-Annexin V and 5 µL of PI at room temperature for 20 min in the dark. After incubation, 300 µL of binding buffer was added, and samples were analysed using a FACS Canto II flow cytometer (BD Biosciences). To avoid lentiviral GFP interference, siRNAs targeting ASH2L were used instead of shRNA for this analysis.

### Orthotopic tumour xenograft

The Animal Care and Use Committee of Hanyang University approved all the mouse trials (Seoul, Republic of Korea). Five-week-old female NOD/SCID mice were purchased from KOATECH (Pyeongtaek-si, Republic of Korea). For the limiting dilution assay, serially diluted MCF7 cells stably overexpressing ASH2L (ASH2L) or control vector (CON) (5000–20,000 cells) and HCC1428 cells expressing either control shRNA (shCON) or ASH2L shRNA (shASH2L) (10,000–40,000 cells) were resuspended in Matrigel (BD Biosciences) and orthotopically injected into the mice (*n* = 6 per group). One week prior to cell injection, E2 pellets (0.72 mg/pellet; 60-day release; Innovative Research of America) were implanted into mice. The frequency of tumour-initiating cells (TIC) was analysed using the L-Calc software (STEMCELL Technologies, Vancouver, Canada, http://www.stemcell.com). Mice with tumour volumes of 30 mm³ or greater were considered as tumour-bearing. For the animal experiments using tamoxifen or SCH772984, control or ASH2L-overexpressing MCF7 (4 × 10^6^ cells) or shcontrol or ASH2L-knockdown ZR-75-1 cells (1 × 10^7^ cells) were orthotopically injected into the mice (*n* = 8 per group) implanted with E2 pellets (0.72 mg/pellet; 60-day release; Innovative Research of America). When the tumour volume reached ~100 mm^3^, subcutaneous injections of tamoxifen pellets (5 mg/pellet, 60-day release) and/or intraperitoneal injections of SCH772984 (10 mg/kg) were injected for 2–3 weeks (once daily for 18 days). Tumour volumes were measured twice per week with calipers and calculated as 1/2 × (long diameter) × (short diameter)^2^.

### Statistical analysis

The unpaired Student’s *t*-test was used to determine statistical significance between the two groups. One-way analysis of variance (ANOVA) with a post-hoc least significant difference (LSD) test (equal variance) or Tukey test and repeated-measures (RM) ANOVA with a post-hoc LSD test were used to assess multiple group comparisons and repeated measures. Statistical significance for the limiting dilution assay was evaluated using the Chi-square (Deviance) test. Tumour-free survival was analyzed by the Kaplan–Meier method and compared using the log-rank (Mantel–Cox) test. For survival analysis, the Kaplan–Meier method with log-rank test and Cox proportional-hazards model were used. *r* values were calculated using Pearson’s correlation coefficient. All *p*-values were two-sided and calculated by SPSS (SPSS Inc., Chicago, IL). *p* < 0.05 was considered statistically significant.

## Results

### Abnormal ASH2L expression is associated with worse prognosis and tamoxifen resistance in ER-positive breast cancer patients

**Fig. 1:**
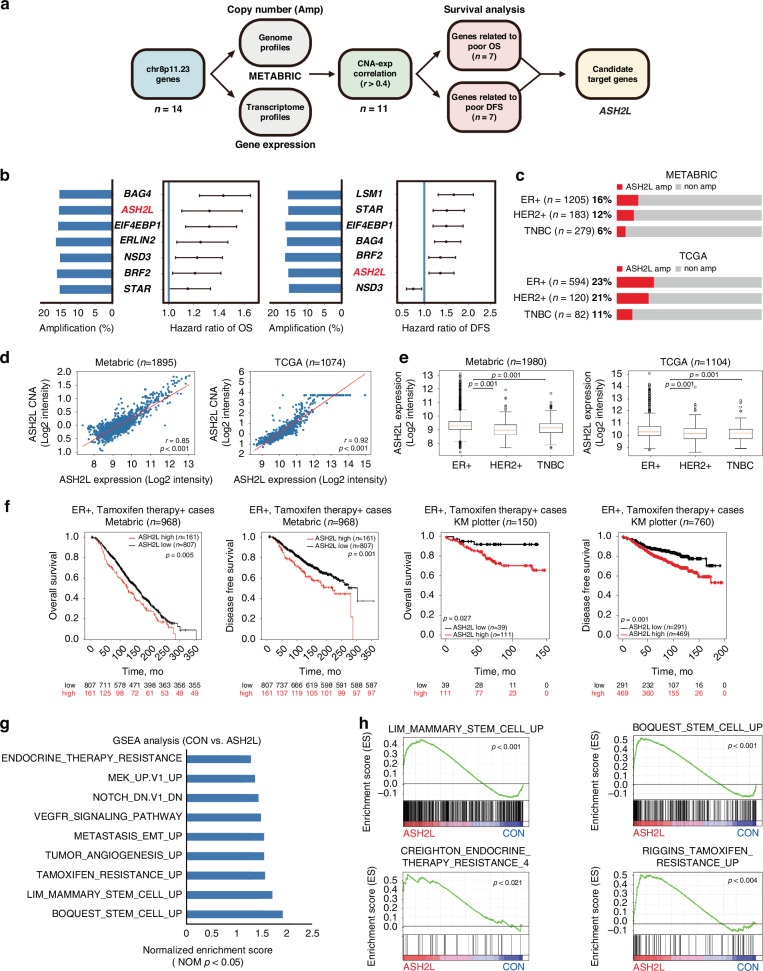
ASH2L is associated with poor prognosis in ER-positive breast cancer. **a** Schematic diagram depicting the process to identify putative target genes within chr8p11.23 in the METABRIC dataset. **b** Forest plot showing the hazard ratios of overall survival (OS) and disease-free survival (DFS) of candidate target genes. Amplification frequencies are represented as bar graphs. **c** Oncoprints depicting ASH2L genomic changes in the relevant breast cancer datasets. **d** Scatter plots displaying the correlation between mRNA expression and copy number alteration (CNA) of ASH2L in the METABRIC (left panel) and TCGA (right panel) datasets. The Pearson’s correlation coefficient was used to determine the *r* value. One-way ANOVA with post-hoc LSD test was used to generate *p*-values. **e** Box plots displaying the levels of ASH2L expression in the specified breast cancer subtypes from the METABRIC and TCGA datasets. Vertical bars (whiskers) = lowest and highest values, horizontal lines (red) inside the boxes = medians, box = 25–75th percentiles. *p*-values were calculated by one-way ANOVA with post-hoc Tukey test. **f** Analysis of the OS and DFS for ER-positive breast cancer patients treated with tamoxifen in the METABRIC and Kaplan Meier-plotter datasets according to the expression level of *ASH2L*. *P*-values were determined using the log-rank test and the Kaplan-Meier method with the log-rank test. **g**, **h** Gene set enrichment analysis (GSEA) of the differentially expressed genes (DEGs) identified by RNA-seq data demonstrates the enrichment of the indicated gene signatures in ASH2L-overexpressing cells (ASH2L) in comparison to the control cells (CON). The adjusted *p*-value (*q*-value), which measures the false discovery rate, was computed to assess statistical significance.

Chromosome 8p11.23 has been known to be amplified in various types of cancer [20]. To discover potential candidate genes with oncogenic roles within chr8p11.23, we first sorted the chr8p11.23 genes with high amplification frequencies (>15%) in ER-positive breast cancer using the Molecular Taxonomy of Breast Cancer International Consortium (METABRIC) dataset. Among the frequently amplified genes, those with a positive correlation (*r* > 0.4) between mRNA expression and copy number alterations were selected. Survival analyses were performed to identify putative target genes associated with poor overall survival (OS) and disease-free survival (DFS) in ER-positive breast cancer (Fig. 1a, b). Notably, the lysine methyltransferase nuclear receptor binding SET domain protein 3 (NSD3), which we previously reported as a driver of tumour initiation and metastasis in breast cancer [21], was one of the candidate genes. Among the candidate target genes, we selected absent, small, or homeotic 2-like protein (ASH2L), whose function is associated with histone H3 lysine 4 tri-methylation as a core subunit of MLL histone H3K4 methyltransferase complexes.

First, we examined aberrant copy number alterations of ASH2L in ER-positive breast cancer in the METABRIC and The Cancer Genome Atlas (TCGA) datasets. ASH2L amplification was observed in ~15% of ER-positive breast cancer samples from the METABRIC dataset and 23% from the TCGA dataset, respectively (Fig. 1c). The copy number alteration of ASH2L was correlated with its gene expression (*r* = 0.85, *p* < 0.001, in METABRIC; *r* = 0.92, *p* < 0.001, in TCGA; Fig. 1d). ASH2L expression was the highest in ER-positive breast cancer among three subtypes of breast cancer (*p* = 0.001 in METABRIC; *p* = 0.001 in TCGA; Fig. 1e). Consistently, ASH2L expression was significantly higher in ER-positive than in ER-negative tumours in both METABRIC and TCGA cohorts (*p* < 0.001, in METABRIC; *p* < 0.001, in TCGA; Supplementary Fig. S1a). In ER-positive breast cancers, no significant correlation was observed between ASH2L and estrogen receptor 1 (ESR1) expression (*r* = 0.20, *p* < 0.001, in METABRIC; *r* = 0.16, *p* < 0.001, in TCGA; Supplementary Fig. S1b). We next examined whether ASH2L affects ERα expression or transcriptional activity. In multiple ER-positive breast cancer cell lines (MCF7, T47D, MCF7-TamR, HCC1428, and ZR-75-1), ASH2L overexpression or knockdown did not alter ERα mRNA or protein levels, nor did it affect ERE (estrogen response element)-luciferase activity (Supplementary Fig. 1c, d). We further examined the clinical relevance of ASH2L using human primary breast cancer datasets. In the METABRIC cohort, high ASH2L expression was associated with worse OS and DFS in ER-positive breast cancer (OS, *p* = 0.002; DFS, *p* = 0.003; Supplementary Fig. S1e) as well as in patients who had treatment with tamoxifen (OS, *p* = 0.005; DFS, *p* = 0.001; Fig. 1f). Analysis of breast cancer patient survival using the Kaplan-Meier plotter (KM plotter) dataset showed that ASH2L overexpression was associated with poorer survival outcomes in patients with ER-positive breast cancer (*p* = 0.027; DFS, *p* < 0.001; Supplementary Fig. S1e) and those who underwent hormonal therapy (OS, *p* = 0.027; DFS, *p* = 0.001; Fig. 1f). Collectively, these data suggest that ASH2L is a prognostic factor for poor survival and tamoxifen resistance in ER-positive breast cancer.

To further study the molecular mechanisms underlying the poor prognosis caused by high ASH2L expression, we performed RNA sequencing (RNA-seq) analysis of ASH2L-overexpressing MCF7 cells. Gene set enrichment analysis (GSEA) of the RNA-seq data revealed that the differentially expressed genes were significantly enriched in many important cancer-related pathways, including tumour metastasis, cancer stem cells (CSCs), and tamoxifen resistance (nominal *p* < 0.05; Fig. 1g, h). These results suggest that ASH2L is involved in tamoxifen resistance and CSC-like properties.

### ASH2L induces tamoxifen resistance and promotes CSC-like phenotypes in ER-positive breast cancer

**Fig. 2:**
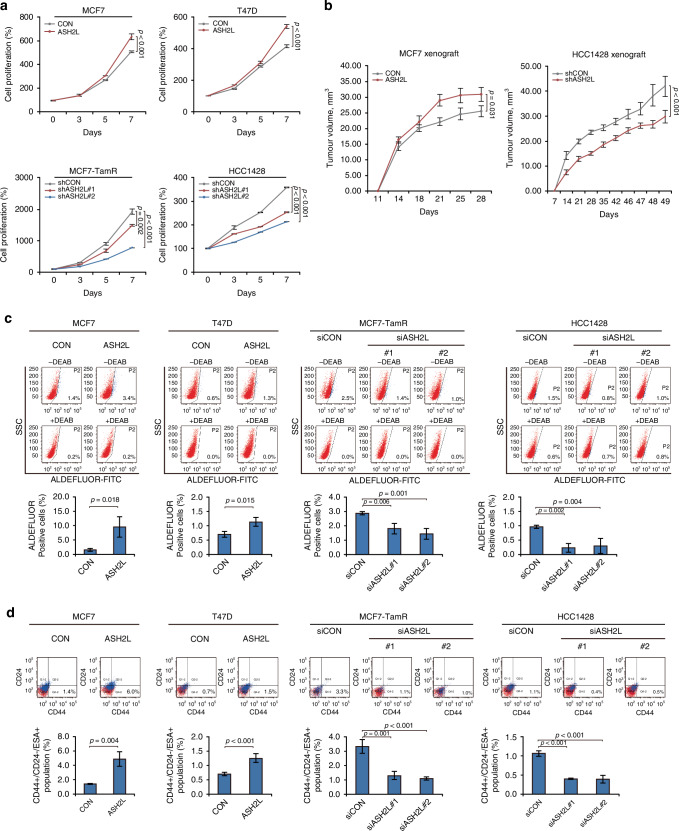

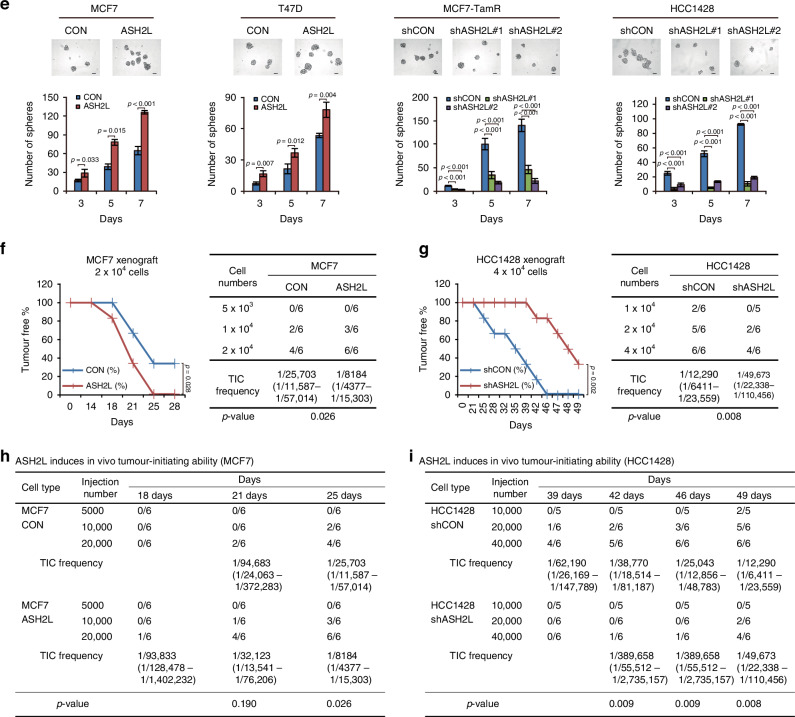
ASH2L promotes a CSC-like phenotype in ER-positive breast cancer. **a** Cell viability assays in the indicated cell lines with overexpression (ASH2L) or knockdown (shASH2L) of ASH2L expression. Data are shown as mean ± SD from *n* = 3 technical replicates. *p*-values by RM ANOVA (MCF7 and T47D) or RM ANOVA with a post-hoc LSD test (MCF7-TamR and HCC1428). **b** Tumour growth curves obtained from orthotopic xenograft mouse models injected with MCF7 cells (1 × 10^4^ cells) or HCC1428 cells (2 × 10^4^ cells). Mean ± standard error of the mean (SEM) (*n* = 6 per group). *p*-values by RM ANOVA. **c** ALDEFLUOR assay was performed to analyse ALDH-high populations in the ER-positive breast cell lines that had ASH2L overexpression (ASH2L) or were temporarily knocked down (siASH2L) by ASH2L siRNA. Data represents the mean ± SD from *n* = 3 biological replicates. *p*-values from two-sided Student’s *t*-tests (MCF7 and T47D) or one-way ANOVA with a post-hoc LSD test (MCF7-TamR and HCC1428). **d** FACS analysis of CD44+/CD24-/ESA+ cells in breast cancer cell lines with stable overexpression (ASH2L) or transient knockdown of ASH2L (siASH2L). Representative dot plots of CD44-APC versus CD24-PE expression for variable cells (P1gate, red) and for ESA+ cells (P2gate, blue) from P1 gate. Bar graphs indicate the percentage of CD44+/CD24 -/ESA+ cells in the indicated groups. Data are shown as the mean ± SD from *n* = 3 biological replicates. *p*-values from two-sided Student’s *t*-tests (MCF7 and T47D) or one-way ANOVA with a post-hoc LSD test (MCF7-TamR and HCC1428). **e** Tumorsphere formation assays to assess the stemness of the indicated cell lines. Data are shown as the mean ± SD from *n* = 3 biological replicates. *p*-values from two-sided Student’s *t*-tests (MCF7 and T47D) or one-way ANOVA with a post-hoc LSD test (MCF7-TamR and HCC1428). Scale bars = 100 μm. **f**, **g** The percentage of tumour-free mice in the indicated groups of xenograft mice. *p*-values were determined based on the log-rank (Mantel-Cox) test. The table shows the tumour-initiating cell (TIC) frequency analysed using the L-Calc software (Stem Cell Technologies). Statistical significance was evaluated using the Chi-square (deviance) test. For in vivo limiting dilution assays, serially diluted MCF7 cells stably overexpressing ASH2L (ASH2L) or control vector (CON) (5000-20,000 cells) (**h**), and HCC1428 cells expressing either control shRNA (shCON) or ASH2L shRNA (shASH2L) (10,000-40,000 cells) (**i**), were orthotopically injected into the mammary fat pads of NOD/SCID mice. The tumour-initiating cell (TIC) frequency was calculated using L-Calc software (STEMCELL Technologies, Vancouver, Canada, http://www.stemcell.com). *p*-values were calculated using Chi-square deviance test via L-Calc software.

To demonstrate the oncogenic properties of ASH2L, we first investigated whether ASH2L affects the proliferation rate of ER-positive breast cancer cells by performing cell viability assays. Based on the information from the Cancer Cell Line Encyclopedia (CCLE) dataset [22, 23], we identified the expression level of ASH2L in human breast cancer cells and selected appropriate cell lines for the study (Supplementary Fig. S2a). We generated ASH2L-overexpressing cell lines with MCF7 and T47D cells and ASH2L knockdown cell lines using tamoxifen-resistant MCF7 (MCF7-TamR) and HCC1428 cells. The results showed that ASH2L overexpression accelerated the proliferation rate of MCF7 and T47D cells, while shRNA-mediated ASH2L-knockdown reduced the proliferation in both MCF7-TamR and HCC1428 cells (Fig. 2a). Moreover, ASH2L overexpression accelerated, and its knockdown inhibited in vivo tumour growth in orthotopic xenograft mouse models (*p* = 0.031 and *p* < 0.001, respectively; RM ANOVA; Fig. 2b and Supplementary Fig. S2b).

Based on the GSEA results, we tested whether ASH2L affects response to tamoxifen and CSC-like properties in ER-positive breast cancer. ALDEFLUOR assay results showed that ASH2L overexpression expanded CSC-like populations with high ALDH activity in MCF7 and T47D cells, while ASH2L knockdown suppressed these CSC-like phenotypes in MCF7-TamR and HCC1428 cells, which exhibit high endogenous ASH2L expression levels (Fig. 2c). Similar results were observed in FACS analysis of the CD44+/CD24-/ESA+ population, another characteristic marker of breast CSCs (Fig. 2d). Additionally, ASH2L overexpression enhanced the tumour sphere-forming capacity in MCF7 and T47D cells, whereas ASH2L knockdown reduced this capacity in MCF7-TamR and HCC1428 cells, as evidenced by tumour sphere formation assays (Fig. 2e). In vivo limiting dilution assays demonstrated that ASH2L overexpression in MCF7 cells significantly increased tumour-initiating cell (TIC) frequency compared to control cells (1/8184; 95% CI: 1/4377–1/15,303 vs. 1/25,703; 95% CI: 1/11,587–1/57,014; *p* = 0.026; Chi-square deviance test; L-Calc software; Fig. 2f, right; Fig. 2h), whereas ASH2L knockdown in HCC1428 cells reduced TIC frequency (1/49,673; 95% CI: 1/22,338–1/110,456 vs. 1/12,290; 95% CI: 1/6411–1/23,559; *p* = 0.008; Chi-square deviance test; L-Calc software; Fig. 2g, right; Fig. 2i). Consistently, ASH2L-overexpressing MCF7 cells exhibited a significantly lower tumour-free rate, while ASH2L-knockdown HCC1428 cells showed an increased tumour-free rate, indicating enhanced and impaired tumour-initiating capacity (*p* = 0.028 and *p* = 0.002, respectively; log-rank [Mantel-Cox] test; Fig. 2f, g). Taken together, our data indicates that ASH2L overexpression induces CSC-like properties in ER-positive breast cancer.

**Fig. 3:**
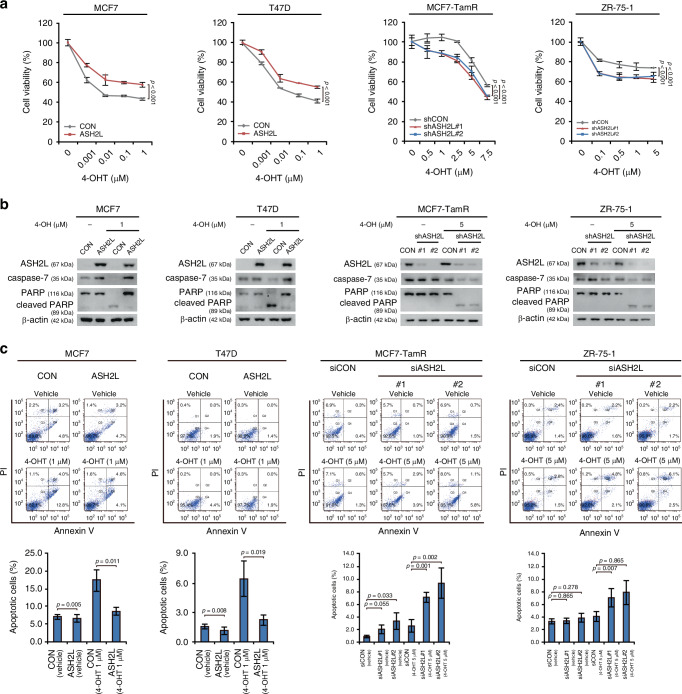

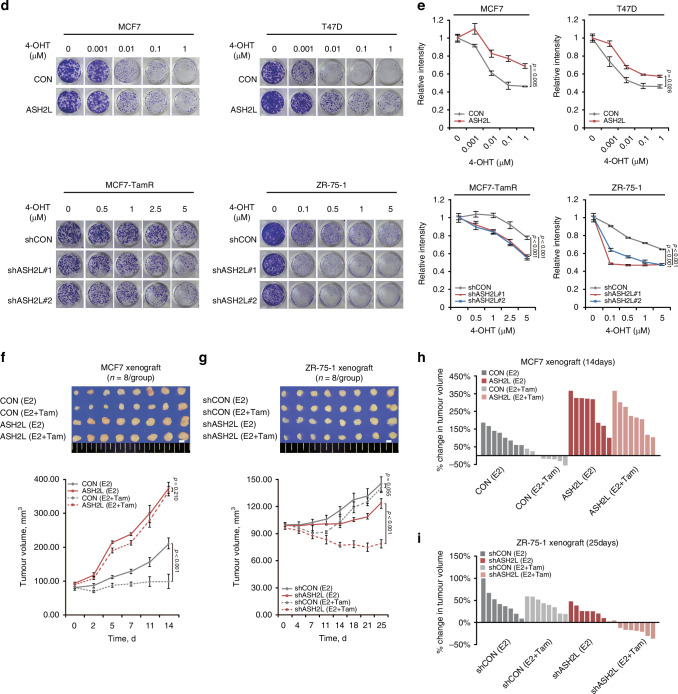
ASH2L induces tamoxifen resistance in ER-positive breast cancer. **a** Cell viability assay was assessed in the specified cell lines treated with 4-hydroxytamoxifen (4-OHT) after 5 days in MCF7, MCF7-TamR, and ZR-75-1 cells, and after 7 days in T47D cells. CON, control (empty vector); ASH2L, ASH2L overexpression; shCON, short hairpin RNA control; shASH2L, ASH2L knockdown. Data are shown as the mean ± SD from *n* = 3 technical replicates. *p*-values by RM ANOVA (MCF7 and T47D) or RM ANOVA with a post-hoc LSD test (MCF7-TamR and ZR-75-1). **b** Immunoblots showing caspase 7 and full length and cleaved PARP in the indicated cells. CON, control (empty vector); ASH2L, ASH2L overexpression; shCON, short hairpin RNA control; shASH2L, ASH2L knockdown. **c** Apoptotic cell populations were analysed by Annexin V/PI staining and flow cytometry in the indicated cell lines that had ASH2L overexpression (ASH2L) or were temporarily knocked down (siASH2L) by ASH2L siRNA. Data represents the mean ± SD from *n* = 3 biological replicates. *P*-values from one-way ANOVA with a post-hoc LSD test. **d** Colony formation assay was performed in the specified cell lines treated with 4-hydroxytamoxifen (4-OHT) for 15 days. CON, control (empty vector); ASH2L, ASH2L overexpression; shCON, short hairpin RNA control; shASH2L, ASH2L knockdown. Data are shown as the mean ± SD from *n* = 3 technical replicates. *p*-values by RM ANOVA (MCF7 and T47D) or RM ANOVA with a post-hoc LSD test (MCF7-TamR and ZR-75-1). **e** Quantification of colony formation in the specified cell lines using ImageJ software after 4-hydroxytamoxifen (4-OHT) treatment for 15 days. Data are shown as mean ± SD from *n* = 3 technical replicates. *p*-values by RM ANOVA (MCF7 and T47D) or RM ANOVA with a post-hoc LSD test (MCF7-TamR and ZR-75-1). **f**, **g** The effect of ASH2L on the response of ER-positive breast cancer cells to tamoxifen in vivo. NOD/SCID mice were orthotopically injected with the control (CON) or ASH2L-overexpressing (ASH2L) MCF7 cells (**f**) and the control (shCON) or ASH2L-knockdown (shASH2L) ZR-75-1 cells (**g**) after implantation of 17β-estradiol (E2) pellets. After the tumour volume reached ~100 mm^3^, the mice were treated with a placebo (E2) or tamoxifen pellets (E2 + Tam), and the tumour sizes of each group were measured for 2–3 weeks. Data are presented as mean [SEM] : CON (E2), 212.22 [16.01]mm^3^; CON (E2 + Tam), 100.35 [21.14]mm^3^; ASH2L (E2), 376.47 [16.38]mm^3^; ASH2L (E2 + Tam) = 366.55 [13.99]mm^3^; shCON (E2), 145.00 [9.60]mm^3^; shCON (E2 + Tam), 140.67 [4.24]mm^3^; shASH2L (E2), 123.60 [5.53]mm^3^; shASH2L (E2 + Tam), 78.79 [4.42]mm^3^). Scale bars = 5 mm. Mean ± SEM (*n* = 8 per group). *p*-values by RM ANOVA with a post-hoc LSD test. Waterfall plots of the percentage change in tumour volumes of individual tumour-bearing mice treated with a placebo (E2) or tamoxifen pellets (E2 + Tam) for 14 days (MCF7 xenograft) (**h**) or 25 days (ZR-75-1 xenograft) (**i**).

We next investigated the impact of ASH2L on tamoxifen resistance in ER-positive breast cancer. The cell viability assay using multiple doses of tamoxifen showed that ASH2L overexpression promoted tamoxifen resistance in the MCF7 and T47D cells (*p* < 0.001 and *p* < 0.001, respectively; RM ANOVA; Fig. 3a). Conversely, knockdown of ASH2L expression in the MCF7-TamR and ZR-75-1 cells enhanced their sensitivity to tamoxifen (*p* < 0.001, *p* = 0.002, and *p* < 0.001, respectively; RM ANOVA with a post-hoc LSD test; Fig. 3a).

To confirm the association between ASH2L and apoptosis reported previously [24, 25], we performed quantitative and qualitative analyses of apoptotic responses following tamoxifen treatment. Caspase 7 and PARP cleavage were assessed by Western blot, showing that ASH2L overexpression reduced tamoxifen-induced apoptosis, whereas ASH2L knockdown enhanced it. Consistently, Annexin V/PI flow cytometry demonstrated a decreased apoptotic cell population in ASH2L-overexpressing cells and and increased population in ASH2L knockdown cells. These results indicate that ASH2L induces anti-apoptosis to tamoxifen treatment (Fig. 3b, c).

To further investigate the effects of ASH2L on tamoxifen response, a colony formation assay was performed and normalised to control wells, and statistical analysis indicated significant differences between experimental groups (*p* = 0.005, *p* = 0.026, and *p* < 0.001, respectively; RM ANOVA; Fig. 3d, e). Consistent with the in vitro results, mice bearing orthotopic xenografts of ASH2L-overexpressing MCF7 cells (n = 8 per group) exhibited resistance to tamoxifen treatment (CON (E2) vs. CON (E2 + Tam), *p* = 0.001; ASH2L (E2) vs. ASH2L (E2 + Tam), *p* = 0.210) along with accelerated tumour growth (CON (E2) vs. ASH2L (E2), *p* < 0.001; RM ANOVA with a post-hoc LSD test; Fig. 3f, h). In ZR-75-1 cells, which exhibited tamoxifen resistance, ASH2L knockdown restored tamoxifen sensitivity in vivo. ASH2L knockdown ZR-75-1 cells (*n* = 8 per group) showed a marked reduction in tumour volume (shCON (E2) vs. shCON (E2 + Tam), *p* = 0.055; shASH2L (E2) vs. shASH2L (E2 + Tam), *p* < 0.001; RM ANOVA with a post-hoc LSD test). Additionally, ZR-75-1 cells with ASH2L knockdown displayed a significantly reduced tumour size compared to control cells (shCON (E2) vs. shASH2L (E2), *p* = 0.008; RM ANOVA with a post-hoc LSD test; Fig. 3g, i). Collectively, these results indicate that ASH2L induces tamoxifen resistance by promoting anti-apoptosis and CSC-like phenotypes in ER-positive breast cancer.

### ASH2L regulates the ITGA6/ERK pathway in an H3K4me3-dependent manner

**Fig. 4:**
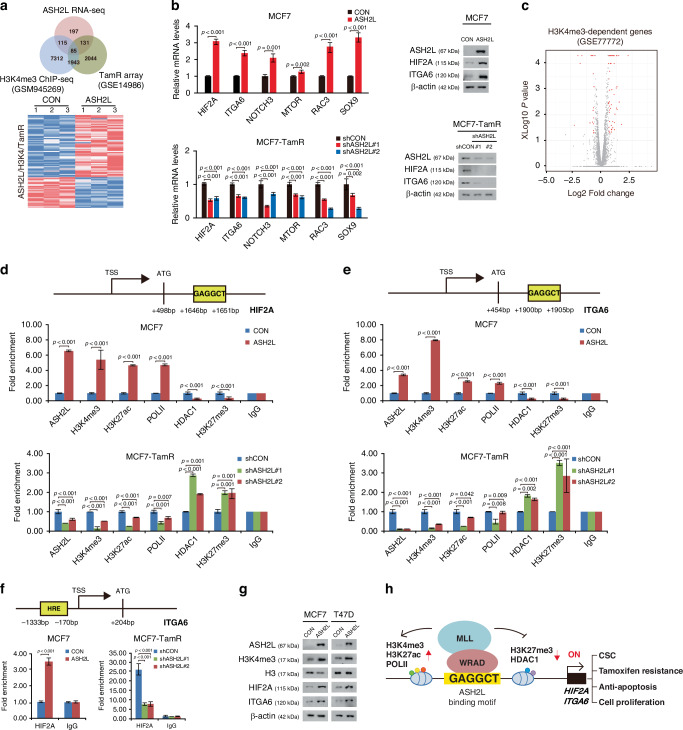
ASH2L activates the HIF2A/ITGA6/ERK pathway in an H3K4me3-dependent manner. **a** Venn diagram showing the numbers of differentially expressed genes (DEGs) from the RNA-seq results shared by the H3K4me3 ChIP-seq data (GSE67790) and the gene expression array data of cell lines that are resistant to Tamoxifen (TamR array, GSE14986). A heatmap showing the differentially expressed genes (DEGs) (≥1.5-fold, *p* < 0.05) between the control (CON) and ASH2L-overexpressing (ASH2L) MCF7 cells (lower panel). **b** qRT-PCR analysis (left panel) and immunoblotting (right panel) were performed in the indicated cell lines to determine the mRNA or protein levels of the target genes, respectively. qPCR data are shown as mean ± SD from *n* = 3 technical replicates. The *p*-values were calculated by a two-sided Student’s *t*-test (MCF7) or a one-way ANOVA with a post-hoc LSD test (MCF7-TamR). **c** Volcano plot showing differentially expressed genes (DEGs) by ASH2L overexpression (red dots) among the H3K4me3-dependent genes (gray dots). *p*-values were calculated using in-house R scripts. Schematic illustration of the HIF2A (**d**) and ITGA6 (**e**) gene’s transcription start site (TSS), which includes the relevant binding motifs (upper panel). The fold enrichment of the histone marks, RNA polymerase II (Pol II), and HDAC1 in the promoter regions of HIF2A (**d**) and ITGA6 (**e**) in response to ASH2L overexpression (middle panel) and ASH2L knockdown (lower panel) was demonstrated by ChIP-qPCR analysis. Data represents the mean ± SD of *n* = 3 technical replicates. The *p*-values were calculated by a two-sided Student’s *t*-test (MCF7) or a one-way ANOVA with a post-hoc LSD test (MCF7-TamR). **f** Schematic illustration of the ITGA6 gene’s transcription start site (TSS) and putative hypoxia response element (HRE) (upper panel). The fold enrichment of the HIF2A in the promoter regions of ITGA6 in response to ASH2L overexpression (lower-left panel) and ASH2L knockdown (lower-right panel) were demonstrated by ChIP-qPCR analysis. Data represents the mean ± SD of *n* = 3 technical replicates. The *p*-values were calculated by a two-sided Student’s *t*-test (MCF7) or a one-way ANOVA with a post-hoc LSD test (MCF7-TamR). **g** Immunoblot of the indicated target proteins in MCF7 cells (left panel) and T47D cells (right panel) overexpressing ASH2L (ASH2L). **h** Proposed model for how ASH2L controls the transcription of the HIF2A and ITGA6 genes.

Because ASH2L is a well-known core subunit of MLL histone H3K4 methyltransferase complexes [26], we compared our RNA-seq data with publicly available histone H3 lysine 4 tri-methylation (H3K4me3) ChIP-seq data (GSM945269) and found that H3K4me3-dependent genes accounted for 38% of the 512 genes whose expression was altered by ASH2L overexpression (Fig. 4a). In addition, we also found that 85 out of the 200 common genes (42%) between the RNA-seq data and ChIP-seq data (GSM945269) were associated with tamoxifen resistance in the comparative analysis of these data with gene expression array data of cell lines that are resistant to tamoxifen (TamR array, GSE14986) (Fig. 4a and Supplementary Table S1). Among these 85 genes, we focused on *EPAS1* (HIF2A), which is associated with tamoxifen resistance and CSCs according to the GSEA results. The altered expression of the ASH2L target genes, including HIF2A and other tamoxifen resistance-related genes, was validated by quantitative reverse transcription PCR (qRT-PCR) and immunoblotting (Fig. 4b). We also identified ASH2L target genes that were closely related to H3K4me3 dependence by comparing our RNA-seq results with another H3K4me3 ChIP-seq dataset (GSE77772) (Fig. 4c and Supplementary Table S2). It has been reported that HIF2A increases the expression of ITGA6 by binding to the hypoxia response element (HRE) region in the ITGA6 promoter [27]. Notably, ITGA6 was identified as a Differentially Expressed Gene (DEG) in the RNA-seq analysis. ITGA6 is a known upstream component that activates the MEK/ERK signalling pathway, which is recognised as a critical mechanism for the development of tamoxifen resistance [28, 29]. Of note, we found that HIF2A and ITGA6 both have the ASH2L-binding motif ‘GAGGCT’ in their promoter regions (Fig. 4d, e). In accordance with these findings, the ChIP-qPCR analysis showed that ASH2L overexpression enhanced the enrichment of H3K4me3, histone 3 lysine 27 acetylation (H3K27ac), and RNA polymerase II (POLII), and dissociation of histone deacetylase 1 (HDAC1) and histone 3 lysine 27 tri-methylation (H3K27me3) at the promoter regions of both HIF2A and ITGA6 in the MCF7 cells, suggesting the transcriptional activation of these genes. Conversely, the opposite enrichment pattern was observed in the ASH2L-knockdown MCF7-TamR cells (Fig. 4d, e). Additionally, our results also showed that changes in ASH2L expression affect HIF2A enrichment at the ITGA6 promoter in MCF7 and MCF7-TamR cells. (Fig. 4f).

To further investigate whether these genes were regulated in an H3K4me3-dependent manner, we conducted immunoblot analysis. Consistent with the ChIP-qPCR results, ASH2L overexpression induced the elevated expressions of HIF2A, ITGA6, and H3K4me3 in MCF7 and T47D cells (Fig. 4g). Taken together, these data indicated that ASH2L directly binds to its binding motifs in the HIF2A and ITGA6 promoters and subsequently epigenetically upregulates the transcription of HIF2A and ITGA6 in an H3K4me3-dependent manner (Fig. 4h).

### H3K4me3-dependent regulation of the ITGA6/ERK signalling pathway by ASH2L enhances endocrine therapy resistance and CSC-like properties

Our data showed that ASH2L regulates the transcription of HIF2A and ITGA6 in an H3K4me3-dependent manner. ITGA6 has been shown to activate the MAPK/ERK pathway in breast cancer, which subsequently regulates the expression of downstream genes such as c-MYC, Cyclin D1, and BCL family members, all of which are involved in regulating cell cycle progression and self-sufficiency [30, 31]. Next, we investigated whether the ITGA6/ERK signalling pathway is responsible for the enhanced proliferation, activation of CSCs, and endocrine therapy resistance induced by ASH2L.

**Fig. 5:**
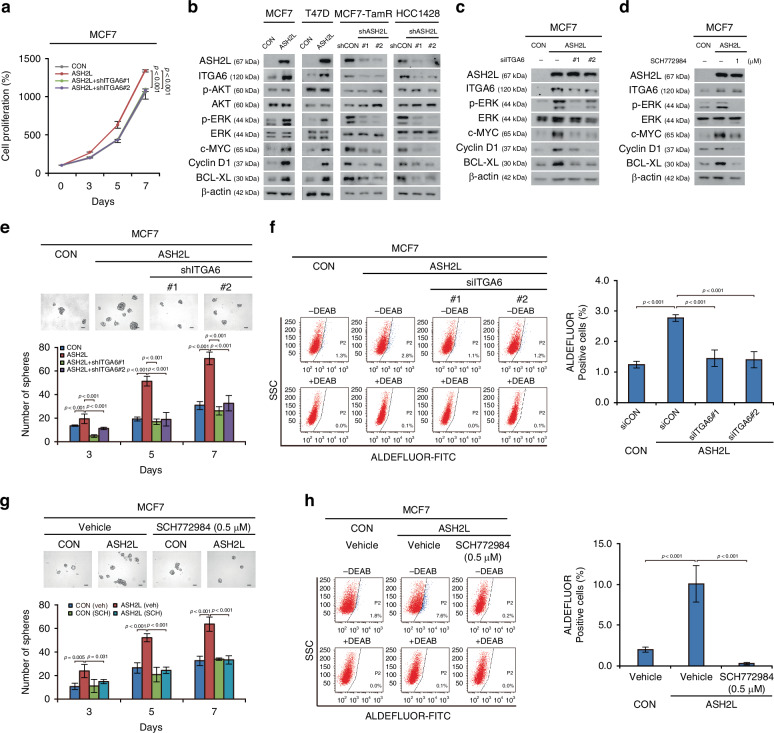
H3K4me3-dependent HIF2A/ITGA6/ERK signalling by ASH2L to promote CSC-like properties. **a** Cell proliferation assay in MCF7 cells with both ASH2L overexpression and ITGA6 knockdown (ASH2L+shITGA6) compared to control MCF7 cells (CON). ASH2L, ASH2L overexpression. Data are shown as the mean ± SD of n = 3 technical replicates. *p*-values were calculated by RM ANOVA with a post-hoc LSD test. **b** Immunoblots showing the protein levels of integrin alpha-6 (ITGA6), ERK, c-MYC, Cyclin D1, and BCL-XL and the phosphorylation levels of ERK in the indicated cells. CON, control (empty vector); ASH2L, ASH2L overexpression; shCON, short hairpin RNA control; shASH2L, ASH2L knockdown. **c** Immunoblot to evaluate the activation of the ERK signalling pathway in ITGA6 siRNA-transfected ASH2L-overexpressing MCF7 cells. CON, control (empty vector); ASH2L, ASH2L overexpression; siITGA6, small interfering RNA targeting ITGA6. **d** Immunoblot of the indicated cells after treatment with 1 µM of SCH772984 or DMSO (vehicle) for 24 h. CON, control (empty vector); ASH2L, ASH2L overexpression. ASH2L-overexpressing MCF7 cells were subjected to a tumorsphere formation assay (**e**) following stable knockdown of ITGA6 using shRNA (shITGA6) and to an ALDEFLUOR assay (**f**) after transfection with ITGA6 siRNA (siITGA6) for 48 h. ASH2L+shITGA6, ASH2L overexpression and ITGA6-knockdown; siITGA6, small interfering RNA targeting ITGA6; siCON, small interfering RNA control. Scale bars = 100 μm. Data are shown as the mean ± SD of *n* = 3 biological replicates. *p*-values were calculated using one-way ANOVA with a post-hoc LSD test. Indicated cells after treatment with either 0.5 µM of SCH772984 or DMSO (vehicle) for 24 h were subjected to a tumorsphere formation assay (**g**) and ALDEFLUOR assay (**h**). CON control (empty vector), ASH2L ASH2L overexpression, veh vehicle, SCH SCH772984. Scale bars = 100 μm. Data are shown as the mean ± SD of *n* = 3 biological replicates. *p*-values were calculated using one-way ANOVA with a post-hoc LSD test.

shRNA-mediated ITGA6 knockdown reversed the proliferation rate promoted by ASH2L overexpression in MCF7 cells (*p* < 0.001; RM ANOVA with a post-hoc LSD test; Fig. 5a). Immunoblotting showed that the expression levels of ITGA6, phosphorylated ERK, and the downstream target genes (c-MYC, Cyclin D1, and BCL-XL) were increased in the ASH2L-overexpressing MCF7 and T47D cells but decreased in the ASH2L-knockdown MCF7-TamR and HCC1428 cells (Fig. 5b). Based on our data that ASH2L is associated with anti-apoptosis (Fig. 3b, c), we examined the expression of apoptotic markers BCL-XL, Bad, and Bax in the indicated cell lines. ASH2L overexpression increased the level of BCL-XL, an anti-apoptotic marker and a downstream gene of ERK signalling, whereas knockdown reduced its expression. In contrast, the expressions of Bad and Bax (pro-apoptotic markers) did not change, as they are not direct targets of ERK signalling (Supplementary Fig. 3a) [32, 33]. Although ITGA6 is known to activate the PI3K/AKT pathway [34], no marked changes in AKT phosphorylation were observed in the breast cancer cell lines we analysed (Fig. 5b). Next, we genetically inhibited ITGA6 expression using ITGA6 siRNA and pharmacologically inhibited ERK using an ERK inhibitor (SCH772984). The enhanced ITGA6/ERK signalling observed in the ASH2L-overexpressing cells was effectively suppressed by transient ITGA6 knockdown using siRNA or by treatment with an ERK inhibitor (Fig. 5c, d).

To further determine whether ITGA6/ERK signalling is responsible for AHS2L-induced CSC-like phenotypes, we performed tumorsphere formation and ALDEFLUOR assays in MCF7 cells. The increased tumour sphere-forming ability and CSC-like populations observed in ASH2L-overexpressing MCF7 cells were significantly suppressed by ITGA6-knockdown or treatment with the ERK inhibitor, SCH772984 (*p* < 0.001 for Fig. 5e, f, h; *p* = 0.005, *p* = 0.031, and *p* < 0.001 for 5 g; one-way ANOVA with a post-hoc LSD test; Fig. 5e–h).

**Fig. 6:**
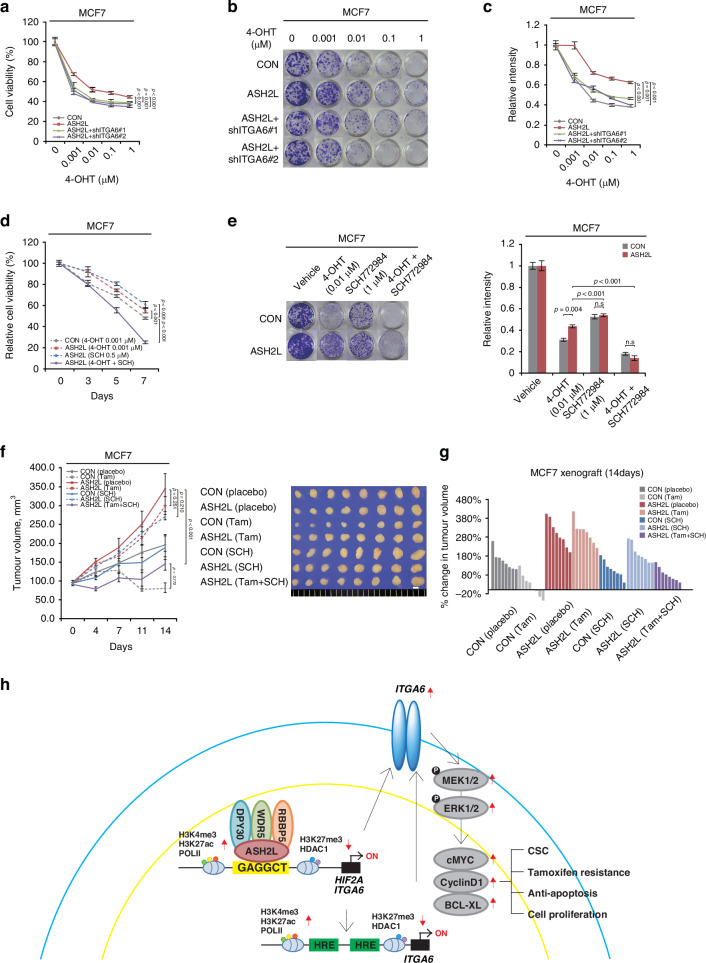
ASH2L induces endocrine therapy resistance through ITGA6/ERK signalling in an H3K4me3-dependent manner. **a**–**c** Cell viability assay (**a**) and colony formation assay (**b**) were performed on the specified cells after treatment with 4-hydroxytamoxifen (4-OHT) for 5 and 15 days, respectively. Colony formation was quantified using ImageJ software (**c**). CON, control (empty vector); ASH2L, ASH2L overexpression; ASH2L + shITGA6, ASH2L overexpression and ITGA6-knockdown. Data are shown as the mean ± SD from *n* = 3 technical replicates. *P*-values were obtained by RM ANOVA with a post-hoc LSD test. **d** Relative cell viability assay in the indicated cells treated with 0.001 µM of tamoxifen (4-OHT), 0.5 µM of SCH772984 (SCH), or a combination of the two (4-OHT + SCH). Data represents the mean ± SD of *n* = 3 technical replicates. *p*-values by RM ANOVA with a post-hoc LSD test. **e** Colony formation experiment in the indicated cells treated with DMSO (vehicle), 0.01 µM of tamoxifen (4-OHT), 1 µM of SCH772984, or the combination of the two (4-OHT + SCH772984) for 15 days. Colony formation was quantified using ImageJ software. Data are shown as the mean ± SD from *n* = 3 technical replicates. The *p*-values were calculated by a two-sided Student’s *t*-test. **f** Tumour growth curves (left panel) and images of the xenograft tumours (right panel) of the orthotopic xenograft mice bearing control (CON) or ASH2L-overexpressing MCF7 cells (ASH2L) upon treatment with the placebo, tamoxifen pellets (Tam), 10 mg/kg SCH772984 (SCH), or a combination of tamoxifen and SCH772984 (Tam+SCH). Data are presented as mean [SEM]: CON (placebo), 199.01 [16.90] mm^3^; CON (Tam), 81.97 [12.44] mm^3^; ASH2L (placebo), 343.88 [41.63] mm^3^; ASH2L (Tam) = 299.93 [23.88] mm^3^; CON (SCH), 190.86 [34.08] mm^3^; ASH2L (SCH), 272.55 [12.12] mm^3^; ASH2L (Tam + SCH), 146.54 [18.47] mm^3^). Scale bars = 5 mm. Mean ± SEM (*n* = 8). *p*-values by RM ANOVA with a post-hoc LSD test. **g** Waterfall plot of the percentage change in tumour volumes of the individual mice in each group treated with the placebo, tamoxifen pellets (Tam), 10 mg/kg SCH772984 (SCH), or a combination of tamoxifen and SCH77294 (Tam + SCH) for 14 days. **h** ASH2L increases the enrichment of H3K4me3, H3K27ac, and PolII recruitment while decreasing HDAC1 and H3K27me3 levels at the HIF2A and ITGA6 promoter regions. The increased HIF2A expression by ASH2L also promotes ITGA6 transcription by binding to the hypoxia response element (HRE) in the promoter region of ITGA6, leading to activation of the ERK pathway. In conclusion, ASH2L-induced tamoxifen resistance and tumour initiation in ER-positive breast cancer are mediated by ITGA6/ERK signalling activation.

Next, we tested whether the ASH2L-induced tamoxifen resistance is mediated by ITGA6/ERK signalling. Tamoxifen resistance, enhanced by ASH2L overexpression in MCF7 cells, was reversed by ITGA6-knockdown (Fig. 6a, b). The quantitative analysis of colony formation demonstrated a similar pattern to cell viability assay results and was statistically significant (*p* < 0.001, *p* = 0.001, and *p* < 0.001, respectively; RM ANOVA; Fig. 6c). Compared to the tamoxifen sensitivity observed in control MCF7 cells, ASH2L-overexpressing cells exhibited resistance to both tamoxifen and ERK inhibitor monotherapy. Notably, the combination treatment with tamoxifen and an ERK inhibitor restored tamoxifen sensitivity in ASH2L-overexpressing MCF7 cells (Fig. 6d, e). Consistent with the in vitro data, treatment with an ERK inhibitor alone did not significantly reduce tumour growth in an orthotopic xenograft model using ASH2L-overexpressing MCF7 cells (ASH2L vs. AHS2L (SCH), *p* = 0.210; RM ANOVA with a post-hoc LSD test). In contrast, combined treatment with tamoxifen and the ERK inhibitor suppressed tumour growth compared to either tamoxifen or ERK inhibitor treatment alone (ASH2L (Tam) vs. ASH2L (Tam + SCH), *p* < 0.001; ASH2L (SCH) vs. ASH2L (Tam + SCH), *p* < 0.001; RM ANOVA with a post-hoc LSD test; Fig. 6f, g). Additionally, there was no significant difference in tumour sizes between the control group treated with tamoxifen and the ASH2L-overexpressing group treated with the combination treatment of tamoxifen and the ERK inhibitor (CON (Tam) vs. ASH2L (Tam + SCH), *p* = 0.772; RM ANOVA with a post-hoc LSD test; Fig. 6f, g).

Collectively, these data suggest that ASH2L induces tamoxifen resistance and CSC-like properties through the ITGA6/ERK signalling pathway in an H3K4me3-dependent manner in ER-positive breast cancer. Furthermore, our data suggested that combination treatment with tamoxifen and an ERK inhibitor may be beneficial for ER-positive breast cancer patients with ASH2L overexpression.

## Discussion

In this study, we demonstrated the crucial role of ASH2L in tamoxifen resistance and tumour initiation, and its effect on the prognosis of ER-positive breast cancer. Mechanistically, ASH2L is recruited to the ASH2L-binding motif of the HIF2A and ITGA6 promoters with the enhancement of H3K4me3and H3K27ac and dissociation of HDAC1 and H3K27me3, which transcriptionally induce these genes. In addition, HIF2A binds to the hypoxia response element (HRE) in the ITGA6 promoter, implying that both ASH2L and HIF2A transcriptionally upregulate ITGA6 expression. Increased ITGA6 expression activated ERK and its downstream pathways, thereby inducing tamoxifen resistance and CSC-like phenotypes (Fig. 6h). Thus, ASH2L contributes to hormonal therapy resistance and CSC activity via the ITGA6/ERK pathway in an H3K4me3-dependent manner, suggesting that ASH2L may be a therapeutic target for ER-positive breast cancer.

Here, we report that ASH2L induces tamoxifen resistance and CSC activity in an H3K4me3-dependent manner. Histone modifications are involved in carcinogenesis and can be used as biomarkers in various cancers, including lung, colorectal, prostate, and breast cancer [16]. It has been reported that histone modification marks are associated with resistance to treatments including anti-estrogen therapy and trastuzumab in breast cancer [35–37]. Several studies have shown that H3K4 methyltransferases, including those of the MLL and SMYD families, are prognostic factors for ovarian, lung, and breast cancer [35]. It has also been shown that the H3K4 methyltransferases KMT2A, KMT2B, and SETD1A promote cancer stem cell properties via Wnt/β-catenin signalling by regulating the H3K4me3 levels on the promoters of associated genes in various cancers including colorectal cancer (CRC) and non-small cell lung cancer [38–40]. SETD1A has been reported to upregulate SOX2 expression by increasing H3K4me3 enrichment in the promoter region of the gene, resulting in enhanced tamoxifen resistance and stem cell populations in breast cancer cells [41]. In addition to H3K4 methyltransferases, other epigenetic regulators such as KDM5B, EZH2, DNMTs, and ARID1A have been implicated in endocrine therapy resistance in breast cancer. KDM5B reduces H3K4me3 at tumour suppressor promoters, affecting cell cycle and tamoxifen response, as shown in our previous report [42]. EZH2 and DNMTs suppress ER signalling through H3K27me3 and DNA methylation-mediated silencing of ER target genes such as GREB1 and PTEN, thereby contributing to tamoxifen resistance [43, 44]. Loss of ARID1A disrupts SWI/SNF-mediated chromatin remodelling, altering ER transcriptional programs [45]. These findings indicate that the role of multiple epigenetic regulators in tamoxifen resistance and suggest that ASH2L may function within a broader network of epigenetic modulators that regulate CSC phenotype and tamoxifen resistance. In this study, we revealed that ASH2L regulates tamoxifen resistance and CSC activity in an H3K4me3-dependent manner in ER-positive breast cancer. Our data show that the overexpressed ASH2L protein is recruited to the HIF2A and ITGA6 promoter regions through the DNA-binding motif of ASH2L and upregulates the transcription of these genes by enhancing H3K4me3 enrichment in the promoter regions. Collectively, these results indicate that ASH2L plays a crucial role in the epigenetic regulation of tamoxifen resistance and CSC activity in an H3K4me3-dependent manner in ER-positive breast cancer.

Our data revealed ASH2L expression was significantly higher in ER-positive breast cancers than in ER-negative ones. Among the ER-positive breast cancers, however, no significant correlation was observed between ASH2L and ESR1. Consistently, ASH2L overexpression or knockdown did not alter ERα mRNA levels, protein expression, or ERE-luciferase activity. These findings suggest that ASH2L expression significantly differs by ER status but does not correlate with ESR1 expression in ER-positive breast cancers. In contrast, Qi et al. [46] reported ASH2L knockdown led to decreased ER expression in BT-483 cells, suggesting a possible role for ASH2L in regulating ER expression. However, these findings are based on a single cell line without clinical validation. It may sensitise ER-positive breast cancer to tamoxifen treatment and thereby be associated with a more favourable prognosis. Our findings instead indicate that ASH2L is a poorer prognostic factor with tamoxifen resistance in ER-positive breast cancers, independently of ER expression or activity. ER expression and activity are regulated in a context-dependent manner through multiple complex mechanisms involving transcriptional (GATA3, FOXA1, and SP1) [47, 48], post-translational (PIK3CA-PI3K/AKT, ERK, and Src) [49, 50], and epigenetic regulators (DNA methylation and histone modification enzymes) [51]. Further molecular mechanistic studies are needed to clarify the role of ASH2L with ER expression and activity according to ligand and ER status.

Our data demonstrated that the ITGA6/ERK signalling pathway is crucial in tamoxifen resistance induced by ASH2L in ER-positive breast cancer. ITGA6 (CD49f) is a cell surface protein that regulates cellular adhesion and is involved in tumour initiation and progression, metastasis, angiogenesis, and anticancer drug resistance [52, 53]. In particular, it promotes cancer stemness and drug resistance in several cancers, including glioblastoma, ovarian, hepatocellular carcinoma, lung, head and neck, and breast cancer [54–57]. Notably, ITGA6 is known to activate downstream PI3K/AKT and MEK/ERK signalling pathways, promoting invasion and metastasis [58, 59]. In breast cancer, ITGA6 promotes radiotherapy resistance via the PI3K/AKT and MEK/ERK pathways [28]. ITGA6 also activates the PI3K signalling pathway, thereby promoting invasion and metastasis in pancreatic cancer [60]. It was reported that the transient knockdown of ITGA6 restored the tamoxifen response in breast cancer and enhanced the anticancer action of aminoflavone, thus inhibiting ITGA6-Src-Akt signalling activation [34]. Similarly, our findings demonstrated that ITGA6 induces tamoxifen resistance exclusively through the ERK pathway in ER-positive breast cancer with high ASH2L expression. ERK signalling is involved in tumorigenesis in human cancers, including ovarian and breast cancer [61–63]. It has been suggested that alterations in the ERK pathway increase the cancer stem cell population in breast cancer cells [64]. Additionally, it was demonstrated that tamoxifen resistance is regulated by the ERK and AKT pathways, and is partially restored by ERK inhibitors in ER-positive breast cancer [65]. Consistent with previous reports [66–68], our data showed that tamoxifen resistance was effectively restored by a combination treatment with tamoxifen and an ERK inhibitor, both in vitro and in vivo. There was no mortality, body weight loss, or gross abnormality of toxicity were observed. The SCH772984 of 10 mg/kg was used according to previous reports and was well tolerated, indicating its potential applicability for clinical translation [69, 70]. Together, our data suggests that combination therapy with tamoxifen and ERK inhibitors can improve tamoxifen resistance in breast cancer patients with ASH2L overexpression.

In conclusion, we demonstrated that ASH2L plays a crucial role in inducing tamoxifen resistance and CSCs in ER-positive breast cancer cells via the ITGA6/ERK signalling pathway in an H3K4me3-dependent manner. Furthermore, our data indicates that combination treatment with tamoxifen and ERK inhibitors is an effective therapeutic strategy for ER-positive, tamoxifen resistant breast cancer patients with ASH2L overexpression and suggests that ASH2L may be a prospective therapeutic target in ER-positive breast cancer.

## Supplementary information

Supplementary Information

Supplementary Figure 1

Supplementary Figure 2

Supplementary Figure 3

Supplementary Table S1

Supplementary Table S2

## Data Availability

The RNA-seq data has been deposited to the Gene Expression Omnibus (GEO) database under the accession number PRJNA946945.
