# Supplementary material for: ASH2L induces tamoxifen resistance via H3K4me3 dependent ITGA6/ERK signaling in ER-positive breast cancer
Source: Br J Cancer. 2026 Feb 24;134(8):1150–65. doi: 10.1038/s41416-026-03347-8 (PMC13036019; doi:10.1038/s41416-026-03347-8)
Supplement: Supplementary file 1 — Supplementary Information [file 41416_2026_3347_MOESM1_ESM.docx]

**SUPPLEMENTARY INFORMATION**

**ASH2L induces tamoxifen resistance via H3K4me3 dependent ITGA6/ERK signaling in ER-positive breast cancer**

Young-Hyeon Kye^1,†^, So-Jeong Moon^1,†^, Hea-Ry Cha^1^, Tack-Hoon Kim^2^, Jeong-Yun Eom^3^, Jae-Kyung Myung^4^, Gu Kong^1, 4,*^

^1^Department of HY-KIST Bio-convergence, College of Medicine, Hanyang University, Seoul, Republic of Korea

^2^Medicinal Materials Research Center, Korea Institute of Science and Technology, Seongbuk-gu, Seoul, Republic of Korea

^3^Department of Pathology, College of Medicine, Hanyang University Hospital, Seoul, Republic of Korea

^4^Department of Pathology, College of Medicine, Hanyang University, Seoul, Republic of Korea

^†^These authors contributed equally to this work.

**SUPPLEMENTARY METHODS**

**Cell Culture and Reagents**

HEK293T cells were obtained from the American Type Culture Collection (ATCC, Manassas, VA, USA). MCF7, T47D, ZR-75-1, and HCC1428 cell lines were purchased from the Korean Cell Line Bank. Tamoxifen-resistant MCF7 cells (MCF7-TamR) were gifted by Prof. Keon Wook Kang (Seoul National University, Seoul, Korea). The MCF7, T47D, and 293T cells were cultured in Dulbecco’s Modified Eagle’s Medium (DMEM; Welgene, Daegu, Republic of Korea) supplemented with 10 % fetal bovine serum (FBS), and the ZR-75-1 and HCC-1428 cells were cultured in Roswell Park Memorial Institute (RPMI; Welgene, Daegu, Republic of Korea) supplemented with 10 % FBS at 37 ℃ in a 5 % CO2 atmosphere. 4-hydroxytamoxifen was acquired from Sigma-Aldrich (St. Louis, MO, USA) and used to treat ER-positive breast cancer cells. For pharmacological inhibition of ERK signaling, SCH772984 was purchased from MedChem Express.

**Transfection of siRNAs and cDNA**

siRNAs against ASH2L (#1, 5’-GUAUGAACGGGUUUUGUUAtt-3,’ cat no. 9070-1; #2, 5’-GUGACUUGUUAUCCUACUAtt-3,’ cat no. 9070-2), were obtained from Bioneer (Daejeon, Republic of Korea), and used with Lipofectamine 2000 (Invitrogen, Carlsbad, CA, USA), as recommended by the manufacturer, to transfect the cells for 48 hours. For genetic inhibition, siRNAs against ITGA6 (#1, 5’-CUGGAAACAUGGACCUUGAtt-3,’ cat no. 3655-1; #2, 5’-CUCUAGGUACGAUGACAGUtt-3,’ cat no. 3655-2), were obtained from Bioneer (Daejeon, Republic of Korea), and transfected into the cells for 24 hours using Lipofectamine RNAiMax (Invitrogen, Carlsbad, CA, USA).

**Lentiviral infection and stable cell generation**

Human ASH2L cDNA was cloned into a lentiviral pLVX-puro vector (Clontech, Mountain View, CA, USA) and ASH2L shRNAs (catalog no. RHS4430-200224671; clone IDs, shRNA #1: V3LHS_635166; catalog no. RHS4430-200225613; clone IDs, shRNA #2: V3LHS_646148) were inserted into the pGIPZ vector (GE Dharmacon, Lafayette, CO, USA). ITGA6 shRNA (catalog no. RHS4430-200206030; clone IDs: shRNA #1: V2LHS_77134; catalog no. RHS4430-200257598; clone IDs, shRNA #2: V2LHS_326015) was cloned into the pGIPZ vector (GE Dharmacon, Lafayette, CO, USA). Cells transfected with the lentiviral pLVX-puro vector and pGIPZ vector were used as controls for overexpression and knockdown studies, respectively. The lentiviruses harboring ASH2L cDNA, or shRNAs were used to infect each cell line with 6㎍/ml polybrene (Sigma-Aldrich, St. Louis, MO, USA) and 2㎍/ml puromycin (Sigma-Aldrich) to establish stable ASH2L-overexpressing and ASH2L-knockdown cell lines, respectively.

**Sulforhodamine B colorimetric assay**

Sigma-Aldrich provided an in vitro toxicology assay kit (Sulforhodamine B based, TOX6) (St Louis, MO, USA). For the assessment of cell proliferation in response to ASH2L expression, MCF7 (3×10^3^ cells/well), T47D (3×10^3^ cells/well), MCF7-TamR (1×10^3^ cells/well), and HCC1428 (5×10^3^ cells/well) were cultured in DMEM or RPMI media containing 10 % FBS. For tamoxifen treatment, Cells (5×10³ cells/well) were seeded in 96-well plates with DMEM or RPMI containing 10% FBS and treated with 4-hydroxytamoxifen for five days, except T47D cells, which were treated for seven days. For combination treatment, the cells (2×10^3^ cells/well) were grown in DMEM containing 10 % FBS and supplemented with 4-hydroxytamoxifen and/or SCH772984 for five days. The cells were then stained with 0.4 % sulforhodamine B for 30 min after being fixed in trichloroacetic acid (TCA) for an hour at 4 °C. For an Optical Density (OD) measurement at 490 nm, the stained cells were destained with 1 % acetic acid and dissolved in 10 mM Tris.

**Tumor sphere formation assay**

In a 6-well ultra-low attachment surface plate, MCF7 (1×10⁴ cells/well), MCF7-TamR (1×10⁴ cells/well), and HCC1428 (1×10⁴ cells/well) cells were cultured in DMEM-Gluta MAX media (Invitrogen, Carlsbad, CA, USA) with 2 % B27, 20 ng/ml of basic fibroblast growth factor (Pepro Tech, Rocky Hill, NJ, USA), 20 ng/ml of epithelial growth factor, and 4 mg/ml of heparin (Sigma-Aldrich) (Corning, Corning, NY, USA). T47D (5×10³ cells/well) was cultured in a DMEM-Gluta MAX medium supplemented with 2 % B27, 10 ng/mL of bFGF, 10 ng/mL of EGF, and 4 mg/mL of heparin. Sphere creation on specific days was measured and quantified.

**RNA-sequencing and Data Collection**

RNA sequencing was performed using the LAS software (Gimpo, Republic of Korea). The TRIzol reagent (Life Technologies, Carlsbad, CA, USA) was used to extract total RNA from the control and ASH2L-overexpressing MCF7 cells. Skewer ver 0.2.2 was used to trim potential sequencing adapters and low-quality bases in the raw reads, and the STAR ver 2.5 software was used to map the resulting high-quality reads to the reference genome. Cuffquant in Cufflinks ver 2.2.1 was used to convert the mapped reads on the reference genome into gene expression values. Differentially expressed genes between the control and ASH2L-overexpressing MCF7 cells were analyzed using Cuffdiff in the Cufflinks package. Genes whose expression differed by at least 1.5-fold between the ASH2L overexpression and control groups were represented. An FDR-adjusted *P* < 0.05 was considered significant using version 7.1 of the Molecular Signature Database (MsigDB; http://software.broadinstitute.org/gsea/msigdb/index.jsp). Gene set enrichment analysis (GSEA) was carried out to functionally classify the differentially expressed genes within the three groups. To compare the ASH2L target genes identified by RNA-seq, H3K4me3 ChIP-seq (GSM945269) and gene expression microarray profiles of tamoxifen-resistant breast cancer cells (GSE14986) were downloaded from the GEO database and re-analyzed. A volcano plot was drawn using in-house R scripts.

**Chromatin Immunoprecipitation quantitative real-time PCR**

Chromatin Immunoprecipitation quantitative real-time PCR assays were carried out according to the manufacturer’s instructions (Upstate Biotechnology, Lake Placid, NY, USA). Briefly, the cells were crosslinked with 1 % formaldehyde at 37 °C for 10 min and treated with 0.125 M glycine to stop the crosslinking. The cell pellets were sonicated using a Bioruptor and resuspended in a Sodium Dodecyl Surfate (SDS) lysis buffer (Cosmo Bio Co. Ltd., Tokyo, Japan). The cell lysates were then immunoprecipitated with certain antibodies at 4 °C for 4 hours, followed by an additional 2 hours of incubation at 4 °C with salmon sperm DNA coupled to protein A or G-agarose (Millipore). After being cleaned, elution, and reverse-crosslinked with 5 M NaCl, the precipitate was incubated at 65 °C overnight. The DNA fragments were precipitated from the eluate and dissolved in ddH2O. The enrichment of the ChIP signal was analyzed by qPCR (signal/noise ratio) as described above using the following specific primers: HIF2A promoter (5′-ACCGAGAGTGGTTGGGAGA-3′ and 5′-CAGAAGTCTCCACTGCAAAGC-3′) and ITGA6 promoter (5′-CACCTGCTAGGTGAAGCACA-3′ and 5′-GCAGCTGGCCAGGCATATAG-3′; 5’-CTCCCTCGCTCTGTGCTACT-3’ and 5’-GTCTCCAGCTGCCCGGTA-3’).

**Immunoblotting**

The following antibodies were used for immunoblotting or immunofluorescence staining: ASH2L (A300-489A) from BETHYL (Texas, USA); H3K4me3 (ab9049) from Abcam (Cambridge, UK); HIF2A (sc-13596), ITGA6 (sc-374057), CCND1 (sc-8396), ERα (sc-8002), PolII (sc-47701), normal mouse IgG (sc-2025), and normal rabbit IgG (sc-2027) from Santa Cruz (Dallas, TX, USA); Caspase-7 (9492S), PARP (9542S), BCL-XL (2762), cMYC (5605S), pERK (9101S), ERK (9102S), pAKT (4060S), AKT (9272S), Bad (9292S), and Bax (2772S) from Cell Signaling Technology (Beverly, MA, USA); H327ac (06-599), H3K27me3 (07-449), and β-actin (MAB1501R) from Millipore (Billerica, MA, USA).

**Quantitative reverse-transcription PCR**

Total RNA was extracted using Invitrogen's TRIzol reagent, and cDNA was produced from the extracted RNA using Access RT-PCR Systems (Promega, Madison, WI, USA), and then subjected to qRT-PCR to quantify the mRNA expression levels. The qRT-PCR was performed on a CFX Connect Real-Time PCR Detection System (Bio-Rad, Hercules, CA, USA) using SYBR Green dye as recommended by the manufacturer (Applied Biosystems, Foster City, CA, USA), and the data were normalized to the expression of GAPDH. The following primers were used for the qRT-PCR: ESR1, ‘5-CCACCAACCAGTGCACCATT-3’ and ‘5-GGTCTTTTCGTATCCCACCTTTC-3’; HIF2A, 5′-ATGACAGCTGACAAGGAGAAGAA-3′ and ‘5-TGGGCCAGCTCATAGAACAC-3′; ITGA6, 5′-CAGTGGAGCCGTGGTTTTG-3′ and ‘5-CCACCGCCACATCATAGCC-3′; VEGF, 5′-CTGTCTAATGCCCTGGAGCC-3′ and ‘5-ACGCGAGTCTGTGTTTTTGC-3′; NOTCH3, 5′-CCCTTCCTGCGATCAGGACATC-3′ and ‘5-GGTTGCTCAGGCACTCATCCA-3′; mTOR, 5′-GACGAGAGATCATCCGCCAG-3′ and ‘5-ACAAGGGACCGCACCATAAG-3′; SLUG, 5′-AAGCATTTCAACGCCTCCAAA-3′ and ‘5-GGATCTCTGGTTGTGGTATGACA-3′; and GAPDH, 5′-GAAGGTGAAGGTCGGAGTC-3′ and ‘5-GAAGATGGTGATGGGATTTC-3’.

**Luciferase reporter assay**

The ERE-luciferase reporter construct was a gift from Incheol Shin (Hanyang University, Seoul, Korea). For the luciferase reporter assay, cells were plated in 24-well plates and co-transfected with reporter construct and β-galactosidase expression vector for 24 hours using Lipofectamine 2000. For ER-dependent transcriptional activity, the cells were treated with E2 (10 nM) or vehicle with 24 hours of hormone starvation conditions. The luciferase activity was then measured using luciferase assay kit (Promega, Madison, WI, USA) and normalized based on the activity of β-galactosidase to display as relative light unit (RLUs).

**SUPPLEMENTARY FIGURE LEGENDS**

**Supplementary Fig. S1 ASH2L is related to poor survival without ER expression or activity in ER-positive breast cancer.** **a** Box plots displaying the levels of ASH2L expression in ER positive and ER negative breast cancer subtypes from the METABRIC and TCGA datasets. Vertical bars (whiskers) = lowest and highest values, horizontal lines (red) inside the boxes = medians, box = 25–75^th^ percentiles. *p*-values were calculated by one-way ANOVA with post-hoc Tukey test. **b** Scatter plots displaying the correlation between ASH2L and ESR1 mRNA expression in the METABRIC (left panel) and TCGA (right panel) datasets. The Pearson's correlation coefficient was used to determine the *r* value. Two-sided Student’s *t*-tests (METABRIC) or One-way ANOVA with post-hoc LSD test (TCGA) was used to generate *p*-values. **c** qRT-PCR analysis (lower panel) and immunoblotting (upper panel) were performed in the indicated cell lines to determine the mRNA or protein levels of the ESR1, respectively. qPCR data are shown as mean ± SD from n = 3 technical replicates. The *p*-values were calculated by a two-sided Student’s t-test (MCF7 and T47D) or a one-way ANOVA with a post-hoc LSD test (MCF7-TamR, HCC1428, and ZR-75-1). **d** The luciferase reporter assays for ERE in the indicated cell lines in the absence and presence of 10 nM E2 for 24 hours. Data represents the mean ± SD of n = 3 technical replicates. *P*-values by one-way ANOVA with a post-hoc LSD test. **e** Analysis of the overall survival (OS) and disease-free survival (DFS) of ER-positive breast cancer patients in the METABRIC and Kaplan Meier-plotter datasets according to the expression level of *ASH2L. P*-values were determined using the log-rank test and the Kaplan-Meier method. ASH2L = Absent, small, or homeotic 2-like protein; ER = estrogen receptor; METABRIC = Molecular taxonomy of breast cancer international consortium; TCGA = The cancer genome atlas.

**Supplementary Fig. S2 ASH2L expression in ER-positive breast cancer cell lines and in vivo tumor growth.** **a** Immunoblot analysis showing the ASH2L expression levels in the indicated breast cancer cell lines. Quantification of western blots was performed using ImageJ software. Band intensities were normalized to β-actin, and the relative ASH2L expression in T47D, HCC1428, ZR-75-1, and MCF7-TamR cells compared with MCF7 cells. Numbers below each band represent the relative band intensity. **b** Tumor images obtained from orthotopic xenograft mouse models injected with MCF7 cells (1×10⁴ cells, left panel) or HCC1428 cells (2×10⁴ cells, right panel). Scale bars = 5mm.

**Supplementary Fig. S3** **ASH2L regulates anti- and pro-apoptotic markers via ERK signaling.** **c** Immunoblots showing the protein levels of BCL-XL, Bad, and Bax in the indicated cells. CON, control (empty vector); ASH2L, ASH2L overexpression; shCON, short hairpin RNA control; shASH2L, ASH2L knockdown.

**SUPPLEMENTARY TABLES**

**Supplementary Table S1**

List of ASH2L target genes from the ChIP-seq results of H3K4me3 and the TamR array.

**Supplementary Table S2**

List of ASH2L target genes related to H3K4me3 dependent genes.
